# Seroprevalence of Crimean-Congo Hemorrhagic Fever Virus Infection in Humans and Domestic Ruminants, Democratic Republic of the Congo

**DOI:** 10.3201/eid3204.250969

**Published:** 2026-04

**Authors:** Boniface Pongombo Lombe, Yannick Munyeku-Bazitama, Gracia Kashitu-Mujinga, Patrick Kakoni Mukadi, Honoré Mampasi, Curé Georges Mbuyi Tshilenge, Marguerite Pemba Manwana, Noël Mwayakala, Niclette Bibi Zenga, Patient Okitale-Talunda, Elisabeth Pukuta-Simbu, Suguru Taga, Gaston Sefu Amzati, Masahiro Kajihara, Pierre Nsele Mutantu, Jean-Jacques Muyembe-Tamfum, Steve Ahuka-Mundeke, Justin Masumu, Sheila Makiala-Mandanda, Ayato Takada

**Affiliations:** Central Veterinary Laboratory of Kinshasa, Kinshasa, Democratic Republic of the Congo (B.P. Lombe, C.G.M. Tshilenge, J. Masumu); National Pedagogical University, Kinshasa (B.P. Lombe, N. Mwayakala, G.S. Amzati, J. Masumu); University of Kikwit, Kikwit, Democratic Republic of the Congo (Y. Munyeku-Bazitama); University of Kinshasa, Kinshasa (Y. Munyeku-Bazitama, G. Kashitu-Mujinga, P.K. Mukadi, C.G.M. Tshilenge, P. Okitale-Talunda, P.N. Mutantu, J.J. Muyembe-Tamfum, S. Ahuka-Mundeke, S. Makiala-Mandanda); Institut National de Recherche Biomédicale, Kinshasa (Y. Munyeku-Bazitama, G. Kashitu-Mujinga, P.K. Mukadi, M.P. Manwana, N.B. Zenga, P. Okitale-Talunda, E. Pukuta-Simbu, P.N. Mutantu, J.J. Muyembe-Tamfum, S. Ahuka-Mundeke, J.M. Masumu, S. Makiala-Mandanda); Hokkaido University, Sapporo, Japan (Y. Munyeku-Bazitama, P. Okitale-Talunda, S. Taga, M. Kajihara, A. Takada); Institute of Tropical Medicine, Nagasaki University, Nagasaki, Japan (P.K. Mukadi), Compagnie des grands élevages de Katongola, Lubumbashi, Democratic Republic of the Congo (H. Mampasi); University of Zambia School of Veterinary Medicine, Lusaka, Zambia (M. Kajihara, A. Takada)

**Keywords:** viruses, Crimean-Congo hemorrhagic fever, vector-borne infections, zoonoses, CCHF, CCHFV, seroepidemiology, antibody, human, ruminant, Democratic Republic of the Congo

## Abstract

Crimean-Congo hemorrhagic fever virus (CCHFV) was first isolated in the Democratic Republic of the Congo (DRC) in 1956. To date, only 3 sporadic human cases have been reported in the DRC, and data on CCHFV infection in livestock, which are key players in transmission, are scant. We conducted a cross-sectional seroepidemiological study on archived human and animal serum samples collected from 25 provinces across the DRC. Samples were tested using an ELISA detecting CCHFV nucleoprotein-specific antibodies. The seroprevalence of CCHFV infection in humans was 4.4% (55/1,239) and in domestic ruminants was 28.9% (322/1,114). High seroprevalences tended to correlate with increased age, specific climate conditions (e.g., tropical monsoon) and vegetation (e.g., mountain savanna) types, and higher elevation (>600 m). Our findings suggest that CCHFV actively circulates in animals and sporadically transmits to humans in the DRC, highlighting the need for continued surveillance of CCHFV infection.

Crimean-Congo hemorrhagic fever (CCHF) is a severe and potentially fatal human illness caused by CCHF virus (CCHFV), an orthonairovirus belonging to the family Nairoviridae (*1*). CCHFV is mainly transmitted by infected ticks (through bites or crushing) or through direct contact with infected biologic materials (*2*,*3*). Nosocomial and sexual transmission has also been reported (*2*,*3*). CCHFV, formerly known as Congo virus, was first isolated in the blood of a febrile teenage patient and a doctor who had taken care of him in 1956 in Kisangani, Democratic Republic of the Congo (DRC) (*4*–*6*). Soon after, the virus was found to be serologically and morphologically similar to a pathogen causing a disease clinically characterized by fever and hemorrhage occurring among peasants, agricultural workers, and soldiers in the Crimean Peninsula in 1944–1945. Hence the combined name, CCHFV, was proposed and has been used thus far (*7*).

CCHFV infection has been continuously reported in humans, livestock, wildlife, and ticks, becoming the most widely distributed tickborne virus (*6*). It is endemic to Africa, Asia, the Middle East, and the Balkans, and its geographic distribution has been recently expanded to areas believed to be nonendemic in Europe (Italy, France, Portugal, and Spain), where the virus has been detected in humans and ticks (*2*,*8*–*11*). However, although its isolation and the initial human cases were described nearly 70 years ago, reports from the DRC are limited or outdated. Besides the initial human cases, CCHFV has been detected in a 26-year-old man in Biruwe (2008), a city neighboring Kisangani (*12*). Furthermore, only 1 survey conducted in Kamina and Lubumbashi, 2 cities in the southeastern DRC, has documented CCHFV circulation in domestic ruminants (*13*). Little is known about the prevalence of CCHFV infection in domestic animals, risk factors, preferential tick vectors, amplifying hosts, and circulating virus strains (*12*–*15*). Because the DRC often faces hemorrhagic fever outbreaks, including some with unknown causative agents, continuously monitoring pathogens causing hemorrhagic syndromes, such as CCHFV, is essential to enhance preparedness (*16*,*17*).

In this study, we investigated seroprevalence of CCHFV infection in the DRC, focusing on both humans and domestic ruminants. Domestic animals such as cattle, goats, and sheep are often infected with CCHFV without exhibiting major clinical signs (*18*). They harbor the virus and can mount specific antibody responses (*18*). Thus, serosurveys of CCHFV infection in animals are useful for the risk assessment of human infection (*14*,*19*). In this study, we aimed to determine the prevalence of CCHFV-specific antibodies and associated factors in humans and domestic ruminants in the DRC.

## Methods

### Study Design

**Figure 1:**
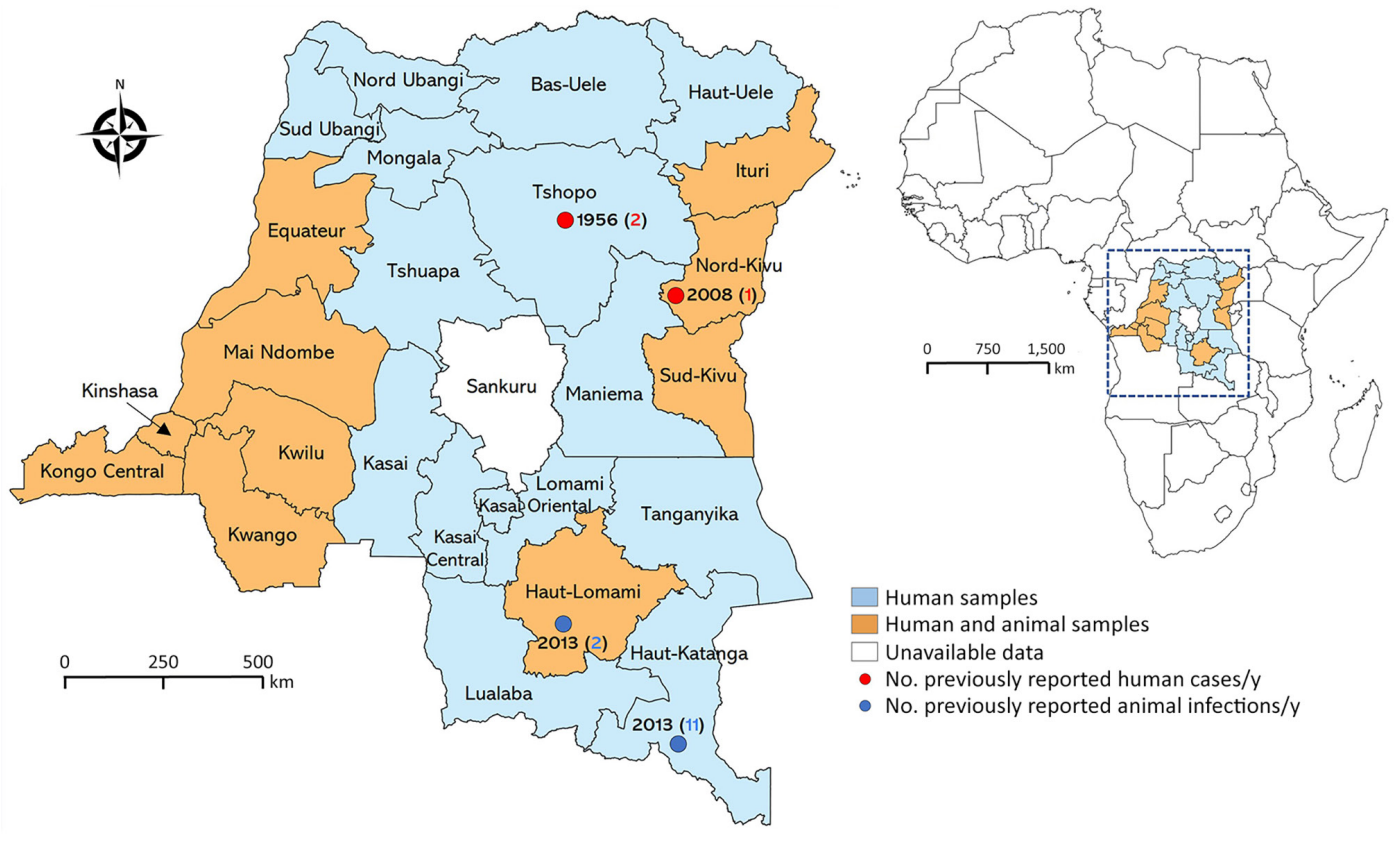
Provinces of sample collection and locations of previously reported human and animal infections in study of seroprevalence of Crimean-Congo hemorrhagic fever virus infection in humans and domestic ruminants, Democratic Republic of the Congo. Light blue shading indicates sites where only human samples were collected; orange shading indicates those where both human and animal samples were collected. Red dots represent locations where human cases have been reported, including number of cases and year of report. Blue dots represent locations with serologic evidence of Crimean-Congo hemorrhagic fever virus in animals, including number of seropositive animals and year of report. Inset shows location of the Democratic Republic of the Congo within Africa. QGIS software version 3.14 (https://qgis.com) was used to generate maps.

**Figure 2:**
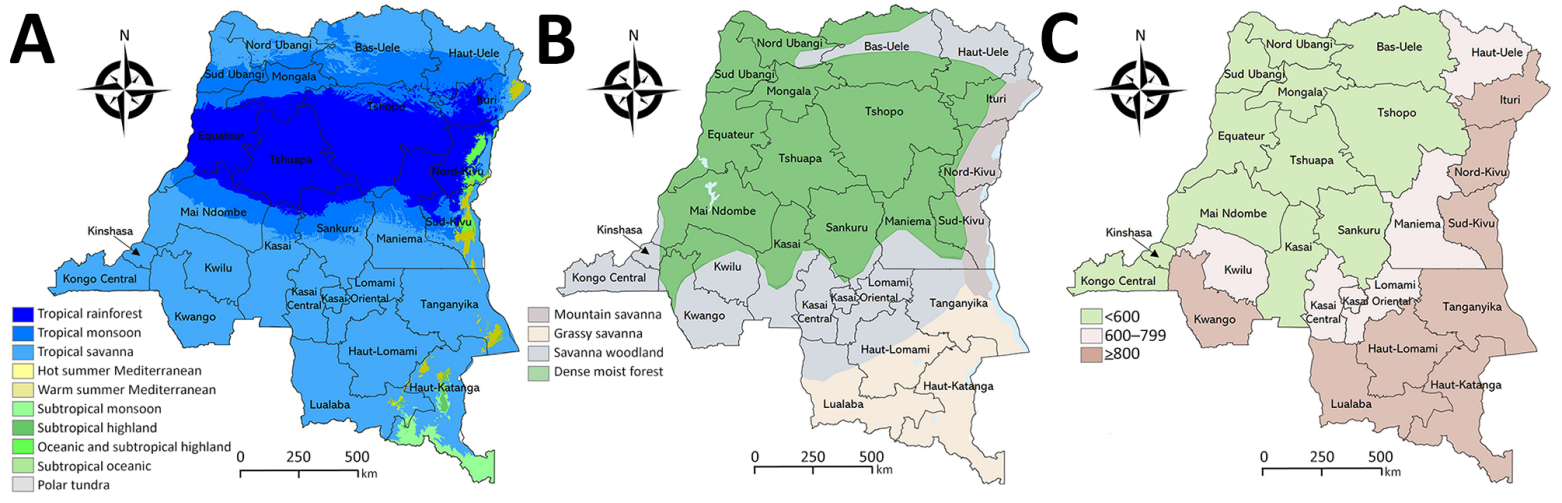
Distribution of provinces by climate, vegetation, and elevation in study of seroprevalence of Crimean-Congo hemorrhagic fever virus infection in humans and domestic ruminants, Democratic Republic of the Congo. A) Climate types based on Köppen-Geiger climate classification (1991–2020 dataset). B) Vegetation type by province. C) Provinces by elevation. QGIS software version 3.14 (https://qgis.com) was used to generate maps.

We conducted a retrospective cross-sectional study using archived human and animal serum samples collected during 2017–2019 in 25 provinces of the DRC (Figure 1). The surveyed provinces represented 3 major DRC climates (tropical savanna, tropical rainforest, and tropical monsoon), 4 main vegetation types (savanna woodland, grassy savanna, dense moist forest, and mountain savanna), and different elevations above sea level (Figure 2) (*20*–*22*).

### Procedures

#### Sample Collection and Processing

Human serum samples were collected in 2017 and 2018 in 25 of the 26 DRC provinces as part of the national yellow fever surveillance system. Of the 1,562 yellow fever–suspected serum samples analyzed at the National Institute for Biomedical Research, 1,492 tested negative using an ELISA for yellow fever virus–specific IgM. Of the samples from that study, we used 1,239 deidentified samples with valid sociodemographic data and sufficient volume for this study. We estimated the minimum required sample size on the basis of an expected seroprevalence of 50% (previous DRC human data were unavailable when the study was conducted and available data on animals were sparse), a precision of 1%, a design effect of 1, and a nonresponse rate of 30%. At least 949 samples had to be included. 

Animal serum samples were collected in 2018 and 2019 from markets and different flocks in commercial and small-holder breeder farms located in 10 provinces. Those samples were primarily collected for seroepidemiological studies targeting common diseases including CCHF, African animal trypanosomiasis, Rift Valley fever, and foot-and-mouth disease. In the sampled provinces, animal samples were collected from multiple farms and flocks. Within a given flock, investigators determined the number of animals to be sampled to ensure the herd's representativeness and that of the area visited. Except for very young animals (<1 month of age) and females in advanced gestation, any animal could be captured and included in the study. We sorted those animal samples to represent different geographic and ecologic patterns related to DRC climate or vegetation, then randomly selected cattle, goat, and sheep samples for this study. The study was approved by the Kinshasa School of Public Health Ethics Committee (ESP/CE/148/2021).

### Variables

The study’s primary outcome was CCHFV serologic status, which could be positive or negative. We used 8 independent variables (sex, age, type of residence, season, climate, vegetation, elevation, and year of sample collection) for the analyses.

### Antibody Detection

We aliquoted serum samples used in this study after collection and stored them in deep freezers for subsequent analyses. We heat-inactivated all serum samples at 56°C for 30 minutes before use (*23*) to inactivate complement in serum while preserving antibody binding activity and for biosafety reasons. We used an in-house CCHFV nucleoprotein (N)–based ELISA to detect CCHFV-specific antibodies (mainly IgG) as described previously (24,25). In brief, we coated Nunc MaxiSorp 96-well ELISA plates (Electron Microscopy Sciences, https://www.emsdiasum.com) with purified N antigen overnight at 4°C then blocked with 3% skim milk in phosphate-buffered saline (PBS) for 1 hour at room temperature. After washing the plates 3 times with PBS containing 0.05% Tween-20 (PBST), we added serum samples diluted (1:100) in PBST containing 1% skim milk and incubated them for 1 hour at room temperature. We then washed the plates >5 times with PBST and detected bound antibodies by using horseradish peroxidase-conjugated protein A/G (Thermo Fisher Scientific, https://www.thermofisher.com) diluted 10,000-fold in 1% skim milk PBST and a 3,3′,5,5′-tetramethylbenzidine substrate. We added 1 normality phosphoric acid to stop the reaction and measured the optical density. We included previously generated mouse N monoclonal antibodies and CCHFV IgG-positive and naive human serum samples as positive and negative controls for assay validation (*24*,*25*). To investigate cross-reactivity with Nairobi sheep disease virus (NSDV), we tested CCHFV N IgG-positive animal samples for NSDV N IgG as described previously using the purified NSDV N antigen in the same ELISA conditions described previously (*24*). We read absorbance at 450 nm using an 800TS Microplate Absorbance Reader (Agilent BioTek, https://www.agilent.com). We carried out assays in duplicate and used the average optical density (OD) value for statistical analyses.

### Statistical Analyses

We analyzed data using Stata version 15.1 (https://www.stata.com) and summarized categorical data using proportions with 95% CIs. We used median and interquartile range (IQR) to summarize continuous variables. Regarding serologic data analysis, we determined the positivity cutoff value using the Smirnov-Grubbs rejection test as described elsewhere; we set the significance threshold at p<0.01 (*26*,*27*). The cutoff OD values were 0.8 for animal samples and 0.95 for human samples. We applied logistic regression to investigate the association between primary outcome and independent variables (sex, age, type of living area, season, climate, vegetation, elevation, and year of sample collection). This preliminary analysis generated estimates of univariable odds ratios (ORs). Next, we performed multivariable logistic regression including all risk factors with the statistical significance threshold set at p<0.05. We used QGIS version 3.16.8 (https://qgis.org) to generate maps.

## Results

### Human Participants, Detection of CCHFV N-Specific Antibodies, and Factors Associated with Seropositivity

Overall, we analyzed 1,239 samples from patients with symptoms suggestive of yellow fever (Table 1; Appendix Table 1). Of those patients, 729 (58.8%) were male and 510 (41.2%) female. The median patient age was 16 (IQR 5–31) years. Most (842 [68.0%]) patients lived in rural areas. Most (906 [73.1%]) samples were collected during the rainy season. Sampling locations differed in climate (tropical savanna, rainforest, and monsoon), vegetation (savanna woodland, grassy savanna, dense moist forest, and mountain savanna), and elevation above sea level (<600, 600–799, and >800 m). Among those categories, the tropical savanna climate (862 [69.6%]), savanna woodland vegetation (596 [48.1%]), and elevations <600 m (860 [69.4%]) were the most represented.

**Table 1:** Sociodemographic characteristics and seroprevalence of Crimean-Congo hemorrhagic fever virus infection in humans in study of seroprevalence in humans and domestic ruminants, Democratic Republic of the Congo*

Variable	No. (%) participants	Seropositivity				OR (95% CI)	p value	aOR (95% CI)	p value
		No.		% (95% CI)					
All participants	1,239	55		4.4 (3.4–5.7)					
Sex									
F	510 (41.2)	21		4.1 (2.7–6.2)		Referent		Referent	
M	729 (58.8)	34		4.7 (3.3–6.4)		1.1 (0.7–1.9)	0.646	1.1 (0.6–1.9)	0.780
Median age, y (IQR)	16 (5–31)								
Age range, y									
0–9	484 (39.1)	13		2.7 (1.6–4.6)		Referent		Referent	
10–19	222 (17.9)	10		4.5 (2.4–8.2)		1.7 (0.7–3.9)	0.211	1.8 (0.8–4.3)	0.165
20–29	214 (17.3)	16		7.5 (4.6–11.8)		2.9 (1.4–6.2)	0.005	3.1 (1.4–6.8)	0.005
30–39	127 (10.2)	4		3.1 (1.2–8.1)		1.2 (0.4–3.7)	0.778	1.2 (0.4–3.9)	0.709
40–49	88 (7.1)	5		5.7 (2.4–13.0)		2.2 (0.7–6.3)	0.148	2.2 (0.8–6.6)	0.139
>50	104 (8.4)	7		6.7 (3.2–13.5)		2.6 (1.0–6.7)	0.046	2.9 (1.1–7.6)	0.027
Type of living area									
Rural	842 (68.0)	41		4.9 (3.6–6.5)		Referent		Referent	
Urban	397 (32.0)	14		3.5 (2.1–5.9)		0.7 (0.4–1.3)	0.286	0.8 (0.3–2.1)	0.662
Season of collection									
Dry season	333 (26.9)	18		5.4 (3.4–8.4)		Referent		Referent	
Rainy season	906 (73.1)	37		4.1 (2.9–5.6)		0.7 (0.4–1.3)	0.318	0.8 (0.4–1.4)	0.453
Climate									
Tropical savanna	862 (69.6)	37		4.3 (3.1–5.9)		Referent		Referent	
Tropical rainforest	283 (22.8)	12		4.2 (2.4–7.3)		0.9 (0.5–1.9)	0.970	0.9 (0.3–2.7)	0.848
Tropical monsoon	94 (7.6)	6		6.4 (2.9–13.5)		1.5 (0.6–3.7)	0.357	1.4 (0.4–5.0)	0.613
Vegetation									
Savanna woodland	596 (48.1)	23		3.9 (2.6–5.7)		Referent		Referent	
Grassy savanna	160 (12.9)	10		6.3 (3.4–11.2)		1.6 (0.8–3.6)	0.193	5.7 (0.7–45.2)	0.101
Dense moist forest	438 (35.4)	19		4.3 (2.8–6.7)		1.1 (0.6–2.1)	0.700	1.2 (0.3–4.1)	0.765
Mountain savanna	45 (3.6)	3		6.7 (2.1–18.7)		1.8 (0.5–6.2)	0.364	6.2 (0.5–69.4)	0.136
Elevation, m									
<600	860 (69.4)	32		3.7 (2.6–5.2)		Referent		Referent	
600–799	90 (7.3)	9		10.0 (5.2–18.1)		2.9 (1.3–6.2)	0.008	3.0 (1.2–7.8)	0.023
≥800	289 (23.3)	14		4.8 (2.9–8.0)		1.3 (0.7–2.5)	0.401	0.4 (0.0–2.8)	0.332
Year of collection									
2017	757 (61.1)	33		4.4 (3.1–6.1)		Referent		Referent	
2018	482 (38.9)	22		4.6) (3.0–6.8)		1.0 (0.6–1.8)	0.864	0.9 (0.5–1.6)	0.680

*aOR, adjusted odds ratio; IQR, interquartile range; OR, odds ratio.

The overall CCHFV seroprevalence was 4.4% (95% CI 3.4%–5.7%). We found an association between seropositivity and age and noted a trend (p = 0.0384 by Cochran-Armitage test for trend) toward increased seropositivity odds with age, especially for persons 20–29 years of age (adjusted odds ratio [aOR] 3.1 [95% CI 1.4–6.8]; p = 0.005) and those >50 years of age (aOR 2.9 [95% CI 1.1–7.6]; p = 0.027). Although results were not statistically significant, samples collected in urban areas or during the rainy season were slightly less likely to test positive. Similarly, samples originating from locations with a predominant tropical rainforest climate were less likely to test positive. Of note, samples from locations with a tropical monsoon climate were 40% more likely to test positive than samples from a tropical savanna climate; however, that association was not statistically significant (aOR 1.4 [95% CI 0.4–5.0]; p = 0.613). Samples from grassy savanna and mountain savanna areas were >5 times more likely to test positive than those from savanna woodland areas, although that difference was not statistically significant (for grassy savanna, aOR 5.7 [95% CI 0.7–45.2], p = 0.101; for mountain savanna, aOR 6.2 [95% CI 0.5–69.4]; p = 0.136). Conversely, samples from areas with an elevation 600–799 meters above sea level were 3 times more likely to test positive (aOR 3.0 [95% CI 1.2–7.8]; p = 0.023).

### Domestic Animals, Detection of CCHFV N-Specific Antibodies, and Factors Associated with Seropositivity

We analyzed a total of 1,114 serum samples from domestic animals, including cattle (706 [63.4%]), goats (357 [32.0%]), and sheep (51 [4.6%]) (Table 2; Appendix Tables 2, 3). Most animals were female (788 [70.7%] vs. 326 [29.3%] male) and <6 years of age (885 [79.4%]). The median age was 4 (IQR 2–5) years. As with human samples, sampling locations differed in terms of climate, vegetation, and elevation above sea level. Study animals originated mainly from areas with a tropical savanna climate (870 [78.1%]) or an elevation of >800 m above sea level (702 [63.0%]).

**Table 2:** Sociodemographic characteristics and seroprevalence of Crimean-Congo hemorrhagic fever virus infection in animals in study of seroprevalence in humans and domestic ruminants, Democratic Republic of the Congo*

Variable	No. (%) animals		Seropositivity			OR (95% CI)	p value	aOR (95% CI)	p value
			No.	% (95% CI)					
All animals	1,114		322	28.9 (26.3–31.6)					
Species									
Other	408 (36.6)		20	4.9 (3.2–7.5)		Referent		Referent	
Cattle	706 (63.4)		302	42.8 (39.2–46.5)		14.5 (9.0–23.3)	<0.001	8.7 (2.3–32.9)	0.001
Sex									
F	788 (70.7)		244	30.9 (27.8–34.3)		Referent		Referent	
M	326 (29.3)		78	23.9 (19.6–28.9)		0.7 (0.5–0.9)	0.019	1.2 (0.8–1.7)	0.333
Median age, y (IQR)	4 (2–5)								
Age range, y									
0–2	291 (26.1)		43	14.8 (11.1–19.3)		Referent		Referent	
3–5	594 (53.3)		189	31.8 (28.2–35.7)		2.7 (1.9–3.9)	<0.001	1.3 (0.8–2.0)	0.226
6–8	143 (12.8)		58	40.6 (32.8–48.8)		3.9 (2.5–6.3)	<0.001	1.6 (0.9–2.8)	0.135
9–12	86 (7.7)		32	37.2 (27.6–47.9)		3.4 (2.0–5.9)	<0.001	1.9 (0.9–3.6)	0.071
Climate									
Tropical savanna	870 (78.1)		201	23.1 (20.4–26.0)		Referent		Referent	
Tropical rainforest	198 (17.8)		86	43.4 (36.7–50.4)		2.6 (1.9–3.5)	<0.001	0.4 (0.2–0.6)	<0.001
Tropical monsoon	46 (4.1)		35	76.1 (61.6–86.2)		10.6 (5.3–21.2)	<0.001	1.3 (0.6–2.7)	0.578
Vegetation									
Savanna woodland	412 (37.0)		23	5.6 (3.7–8.3)		Referent		Referent	
Grassy savanna	240 (21.5)		51	21.3 (16.5–26.9)		4.6 (2.7–7.7)	<0.001	0.2 (0.0–1.0)	0.052
Dense moist forest	10 (0.9)		2	20.0 (5.0–54.1)		4.2 (0.8–21.1)	0.079	1.2 (0.2–9.4)	0.806
Mountain savanna	452 (40.6)		246	54.4 (49.8–58.9)		20.2 (12.8–31.9)	<0.001	0.7 (0.1–3.5)	0.666
Elevation, m									
<600	237 (21.3)		9	3.8 (1.9–7.1)		Referent		Referent	
600–799	175 (15.7)		13	7.4 (4.3–12.4)		2.0 (0.8–4.9)	0.112	2.6 (1.0–6.8)	0.047
≥800	702 (63.0)		300	42.7 (39.1–46.4)		18.9 (9.6–37.4)	<0.001	4.2 (1.3–13.9)	0.019
Year of collection									
2018	301 (27.0)		184	61.1 (55.5–66.5)		Referent		Referent	
2019	813 (73.0)		138	17.0 (14.5–19.7)		0.1 (0.1–0.2)	<0.001	0.3 (0.2–0.5)	<0.001

*aOR, adjusted odds ratio; IQR, interquartile range; OR, odds ratio.

The overall seroprevalence among animals was 28.9% (95% CI 26.3–31.6). That seroprevalence varied among species. The lowest and highest seroprevalence levels were recorded in sheep (2.0% [95% CI 0.3%–12.8%]) (Appendix Table 3) and cattle (42.8% [95% CI 39.2%–46.5] %). Cattle were nearly 9 times more likely to test positive than other species (aOR 8.7 [95% CI 2.3–32.9]; p = 0.001) (Table 2). We noted a trend toward increased seropositivity with age (p<0.0001 by Cochran-Armitage test for trend); animals 6–9 years of age had the highest seroprevalence (40.6% [95% CI 32.8%–48.8%]). However, in multivariable analyses, the association between age and seropositivity decreased both in magnitude and statistical significance. Regarding climate, we recorded the highest seropositivity rate (76.1% [95% CI 61.6%–86.2%]) (Table 2) among animals originating from areas with a tropical monsoon climate. Animals from tropical monsoon climates were 30% more likely to test positive than animals from areas with a tropical savanna climate, although that association was not statistically significant (aOR 1.3 [95% CI 0.6–2.7]; p = 0.578). Of note, animals from areas with mountain savanna vegetation (54.4% [95% CI 49.8%–58.9%]) and those from areas located >800 m above sea level (42.7% [95% CI 39.1%–46.4%]) had high seropositivity rates (Table 2). Odds of seropositivity in animals originating from areas located >600 meters above sea level were >2 times higher.

Because cross-reactivity of orthonairovirus antibodies remains a key issue for serologic analyses using the N antigen of some nairoviruses (*28*,*29*), we sought to distinguish CCHFV-specific antibodies from NSDV, a virus serologically close to CCHFV (*25*,*30*). We retested samples that reacted to the CCHFV N antigen for cross-reactivity to the NSDV N antigen (Appendix Figure). Although some of the CCHFV N IgG-positive serum samples showed appreciable reactivity to NSDV N, OD values were lower than they were to CCHFV N, with 1 exception, indicating that detected antibodies were mostly specific to CCHFV N and cross-reactivity with NSDV was limited.

## Discussion

Investigating CCHFV at the human–animal interface is crucial to learn more about and effectively control CCHF. Animals, particularly domestic ruminants, play an essential role in maintaining and transmitting CCHFV (*14*). In this study, we used domestic ruminants as indicators for the presence of CCHFV infection in the environment and used humans living in the same areas as evidence of ongoing transmission and circulation of the virus. Information is limited on CCHFV circulation among both humans and domestic ruminants across multiple provinces of the DRC, and this study contributes to filling that gap.

In our study, we found an overall seroprevalence of 4.4% (95% CI 3.4%–5.7%) among humans. Similar seroprevalences (4.4%) have been reported among pygmies in Cameroon (*31*) and among farm and wildlife workers (3.9%) in South Africa (*32*). Lower estimates have been reported in Pakistan (2.7%), among blood donors in Mali (1.75%), and among persons living with HIV in the Republic of the Congo (0.6%) (*33*). In contrast, much higher seroprevalence has been reported in Mauritania (11.8%), Tanzania (15.1%), and Uganda (27.0%) (*34*–*36*). Disparities in seroprevalence across surveys might be attributed to the heterogeneity in study populations, sampling strategies, and environmental factors such as climate, vegetation, and elevation. Studies conducted in the general population, including in blood donors, tend to report lower estimates than those targeting populations at high risk that have direct occupational exposure to ticks or studies conducted in locations where environmental factors are favorable for tick development. In fact, our analysis revealed higher seroprevalences in locations characterized by a tropical monsoon climate, a grassy/mountain savanna vegetation, or elevations of >600 meters above sea level, all of which are favorable for tick development (*37*–*41*). Of note, participants living in locations at 600–799 meters above sea level were 3 times more likely to test positive. Conversely, participants from areas located >800 meters above sea level were less likely to test positive. Although those areas are considered to be at high risk for transmission, participants living there might have had lower environmental and occupational exposure to tick bites. Seropositivity significantly increased with age, especially among participants 20–29 years of age and those >50 years of age, who were nearly 3 times more likely to test positive. A similar trend has been reported in other studies (*32*,*34*,*36*,*42*). That finding might reflect cumulative exposure to tick bites, because older participants are more likely to have experienced prolonged or repeated exposure than are younger persons.

CCHFV N-specific antibodies were detected in 28.9% (95% CI 26.3%–31.6%) of animals. That finding is consistent with studies on domestic ruminants in Senegal (32.5%) and Mauritania (33.1%) (*34*,*43*). In contrast, higher seroprevalence estimates have been reported in Cameroon (61.7%) and in Uganda (74.4%), a country neighboring the DRC (*36*,*44*). Surprisingly, a much lower seroprevalence (1.6%) was previously reported in 2 cities in southeastern DRC (*13*). Of note, samples collected in those cities, especially samples from cattle, originated from a single farm; goat and sheep samples were collected from different commercial and private farms within the same city (*13*). Our nearly nationwide study involved multiple sites with diverse ecology, climate, and elevation and used a highly specific assay, which likely resulted in higher seroprevalence estimates. Besides the species composition of the tested animals, differences in the living area or livestock management system, including tick control strategies on farms, might explain the observed variation in seroprevalence. Among tested animals, cattle had a higher seroprevalence (42.8%) than goats (5.3%) and sheep (2.0%). Cattle were nearly 9 times more likely to be seropositive than were other species. A similar trend was also noted in a study from Tanzania (*35*). In most studies conducted in livestock, cattle tend to have the highest seropositivity (*32*,*34*–*36*,*42*,*44*). Higher seroprevalences in larger ruminants such as cattle may be explained by host feeding preferences of ticks. Cattle have been described as suitable hosts for a large number of tick species, including *Hyalomma* spp. (*34*,*45*). In addition, the longevity of larger ruminants could lead to repeated exposure to ticks, increasing the likelihood of persistent infection and sustained antibody responses (*34*).

Male animals were 30% less likely to test positive than female animals in the univariable analysis, corroborating previous studies that have reported sex-related differences in CCHFV infection (*36*,*46*). That finding could be explained by the fact that female animals are kept longer for breeding purposes and milk production on reproductive farms and thereby experience prolonged exposure to tick bites (*34*). Seroprevalence significantly increased with age in the univariable analysis, consistent with earlier reports (*32*,*34*,*36*,*42*). However, the association decreased in magnitude and statistical significance in multivariable analysis. In line with our findings in humans and in previous reports, animal seropositivity was associated with particular climate zones (tropical rainforest and tropical monsoon), vegetation types (grassy savanna and mountain savanna), and elevation above sea level (>800 m) in the univariable analysis. Of note, the association with climate persisted in the multivariable analysis (*37*–*41*).

CCHFV-suitable habitats have previously been shown to be associated with climate and vegetation (grass and shrub cover) that are favorable for tick proliferation (*47*,*48*). Our analysis suggests that the ecologic niche for CCHFV in the DRC might be located in mountainous areas. Indeed, those areas provide grazing lands and climatic conditions suitable for livestock breeding or pasture. Those conditions account for high densities of livestock, especially cattle, which are known to play a critical role in CCHFV transmission (*14*). The mountain climate zone includes the provinces of Ituri, Nord, and Sud Kivu, all of which exhibited high prevalences of CCHFV infection in animals or humans in this study (Appendix Tables 1, 2). Furthermore, because eastern DRC is part of the African Great Lakes region, that area of high CCHFV prevalence is particularly relevant for cattle movement related to breeding and trade (*49*). Cross-border cattle movement might contribute to the spread of tickborne pathogens, particularly through *Hyalomma* ticks, which are capable of surviving under various temperature and humidity conditions (*45*,*49*,*50*). The potential of *Hyalomma* ticks to spread across borders and eventually trigger CCHFV transmission underscores the urgent need for coordinated transboundary disease control strategies. Those strategies should also consider climate change and urbanization as a result of population growth. Both of those factors might act as drivers for CCHFV spread, especially in Africa. Rising temperatures and changing precipitation patterns will contribute to altering the vector ecology, thus establishing *Hyalomma* ticks in new areas.

Because the geographic distributions of CCHFV and some CCHFV-related zoonotic orthonairoviruses, such as NSDV and Dugbe virus (DUGV), might overlap in the DRC, cross-reactivity of N antibodies is a key issue for the serologic diagnosis and surveillance of CCHF. Indeed, our previous study revealed the presence of shared epitopes among the N proteins of CCHFV, NSDV, and DUGV (*24*,*25*). However, those cross-reactive epitopes were not considered to be dominant, because polyclonal antiserum against NSDV and DUGV showed little reactivity with CCHFV N antigens (*24*,*25*). Therefore, those viruses appear to be antigenically distinguishable from each other. Nevertheless, the potential effects of cross-reactivity should not be overlooked in binding assays based on a single antigen. For example, 1 sample exhibited a higher OD value against the NSDV antigen than against the CCHFV antigen (Appendix Figure); thus, NSDV infection cannot be ruled out.

The first limitation of this study is that tick collection for viral genome detection was not performed. A more comprehensive investigation, including CCHFV genome detection from ticks, would provide further insights into the transmission dynamics and phylogeny of strains circulating in the DRC. In addition, the use of samples from febrile patients, although geographically representative, might have introduced selection bias because those patients might have exposure patterns that differ from those of the general population. Future studies should focus on the identified high-risk areas and conduct in-depth analyses of interactions among humans, livestock, and ticks.

In conclusion, we performed a comprehensive investigation of CCHFV infection in the DRC using geographically diverse and representative human and animal samples collected across multiple provinces. Although province-level analyses could not be performed in depth because of small sample sizes, particularly for human samples, the data generated are crucial because they pinpoint regions of interest where CCHFV transmission could be actively occurring and where targeted public health interventions should be initiated. Our study also highlights the potential of leveraging the yellow fever surveillance system as a dual-purpose platform to obtain epidemiologic and clinical data on CCHF, which might not necessarily be associated with a deadly viral hemorrhagic fever. Strengthening this system could enhance preparedness against CCHF outbreaks in the DRC and support transboundary disease control efforts.

Appendix Additional information about seroprevalence of Crimean-Congo hemorrhagic fever virus infection in humans and domestic ruminants, Democratic Republic of the Congo.
