# Supplementary material for: Seroprevalence of Crimean-Congo Hemorrhagic Fever Virus Infection in Humans and Domestic Ruminants, Democratic Republic of the Congo
Source: Emerg Infect Dis. 2026 Apr;32(4):543–52. doi: 10.3201/eid3204.250969 (PMC13094851; doi:10.3201/eid3204.250969)
Supplement: Appendix — Additional information about seroprevalence of Crimean-Congo hemorrhagic fever virus infection in humans and domestic ruminants, Democratic Republic of the Congo. [file 25-0969-Techapp-s1.pdf]

Article DOI: https://doi.org/10.3201/eid3204.250969

EID cannot ensure accessibility for supplementary materials supplied by authors. Readers who have difficulty accessing supplementary content should contact the authors for assistance.

Seroprevalence of Crimean-Congo Hemorrhagic Fever Virus Infection in Humans and Domestic Ruminants, Democratic Republic of the Congo

Appendix

Appendix Table 1. CCHFV seropositivity in humans by province

Province of residence	n	Positive	%
Bas Uele	92	8	8·7
Equateur	51	2	3·9
Haut Katanga	7	0	0·0
Haut Lomami	83	7	8·4
Haut Uele	11	0	0·0
Ituri	7	0	0·0
Kasai	18	0	0·0
Kasai Central	26	1	3·8
Kasai Oriental	1	1	100·0
Kinshasa	342	9	2·6
Kongo Central	48	0	0·0
Kwango	84	1	1·2
Kwilu	29	5	17·2
Lomami	22	2	9·1
Lualaba	57	3	5·3
Maindombe	4	0	0·0
Maniema	1	0	0.0
Mongala	7	1	14·3
Nord Kivu	32	3	9·4
Nord Ubangi	48	2	4·2
Sud Kivu	6	0	0·0
Sud ubangi	23	1	4·3
Tanganyika	13	0	0·0
Tshopo	4	0	0·0
Tshuapa	223	9	4·0

Prevalence estimates for provinces where n < 50 are less accurate to small size

Appendix Table 2. CCHFV seropositivity in animals by province

Province of origin	n	Positive	%
Equateur	3	0	0·0
Haut Lomami	240	51	21·3
Ituri	151	101	67·0
Kinshasa	2	0	0·0
Kongo Central	225	7	3·1
Kwango	10	3	30·0
Kwilu	175	13	7·4
Maindombe	7	2	28·6
Nord Kivu	232	98	42·2
Sud Kivu	69	47	68·1

Prevalence estimates for provinces where n < 50 are less accurate to small size

Appendix Table 3. Detailed demographic characteristics and CCHFV seroprevalence in animals

Species	Variable	Animals		Seropositivity		
		No.	%	No.	%	95% CI
Cattle	All	706	63.4	302	42.8	39.2–46.5
	Sex					
	Female	527	74.6	231	43·8	39.6–48.1
	Male	179	25.4	71	39.6	32.7–47.0
	Age in years					
	0–3	78	11.1	35	44.9	34.2–56.1
	3–6	418	59.2	180	43.1	38.4–47.9
	6–9	126	17.8	55	43.6	35.2–52.5
	9–12	84	11.9	32	38.1	28.3–48.9
Goat	All	357	32.0	19	5.3	3.4–8.2
	Sex					
	Female	231	64.7	12	5.2	2.9–8.9
	Male	126	35.3	7	5.6	2.6–11.3
	Age in years					
	0–3	184	51.5	8	4.3	2.2–8.5
	3–6	156	43.7	8	5.1	2.6–9.9
	6–9	15	4.2	3	20.0	6.3–48.2
	9–12	2	0.6	0	0.0	
Sheep	All	51	4.6	1	2.0	0.3–12.8
	Sex					
	Female	30	58.8	1	3.3	0.4–21.6
	Male	21	41.2	0	0.0	
	Age in years					
	0–3	29	56.9	0	0.0	
	3–6	20	39.2	1	5.0	0.6–30.4
	6–9	2	3.9	0	0.0	
	9–12	0	0.0	0	0.0	

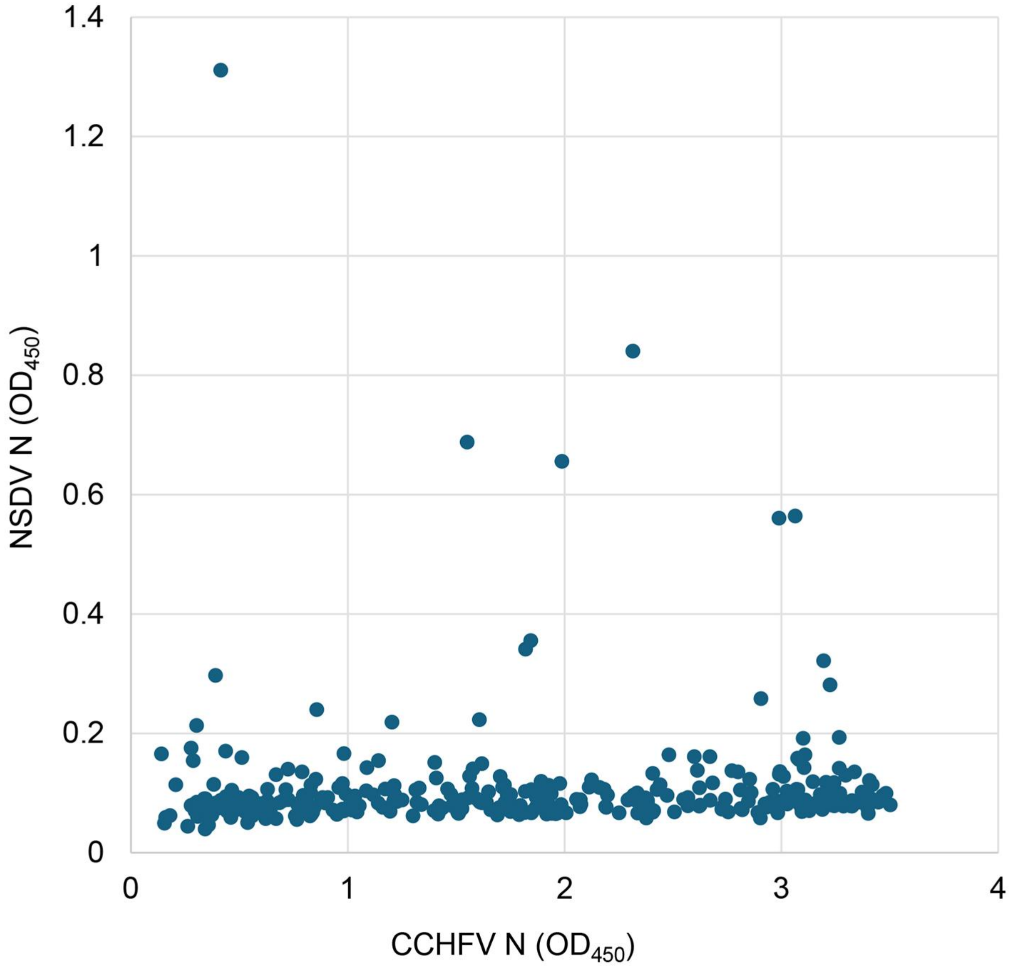

Appendix Figure. Reactivity of tested animal samples to CCHFV and NSDV N. The scatter plot represents reactivity data to CCHFV and NSDV N antigens in ELISA. Limited reactivity was observed with very few outliers above the OD value of 0.4. One sample exhibited an exceptionally high OD value against NSDV N, suggesting that NSDV infection in this animal cannot be ruled out.
